# Assessing the efficacy of electronic quail callers in attracting stubble quail and non-target predators

**DOI:** 10.1371/journal.pone.0271893

**Published:** 2022-07-22

**Authors:** Mia Ray, John G. White, Michael A. Weston, Anthony R. Rendall, Simon D. Toop, Heath Dunstan, Jordan O. Hampton, Raylene Cooke

**Affiliations:** 1 School of Life and Environmental Sciences, Faculty of Science, Engineering and the Built Environment, Deakin University, Burwood, Victoria, Australia; 2 Game Management Authority, Melbourne, Victoria, Australia; 3 Faculty of Veterinary and Agricultural Sciences, University of Melbourne, Parkville, Victoria, Australia; Sichuan University, CHINA

## Abstract

Hunting is a prominent feature of many human societies. Advancements in hunting technologies can challenge the ethics and sustainability of hunting globally. We investigated the efficacy of an electronic acoustic lure (‘quail caller’), in attracting the otherwise difficult-to hunt stubble quail *Coturnix pectoralis* in Victoria, Australia. Using distance sampling, the density and abundance of stubble quail was estimated at 79 sites across a range of habitat types in an agricultural setting, each with an active ‘quail caller’ station continuously broadcasting for 48 hours, and a control station (no broadcast). Quail detectability at the active stations (62.9%) far exceeded that at control stations (6.3%). Most (57%) detections occurred within 30 m of active ‘quail callers’. Stubble quail relative abundance was substantially greater when ‘quail callers’ were broadcasting. Cameras mounted near ‘quail callers’ identified the predatory red fox as a non-target predator, although rates of attraction appear similar between active and control sites. ‘Quail callers’ are highly effective at attracting stubble quail and concentrating them to a known area, raising questions in relation to sustainable hunting practices, indirect effects, and ethical implications. ‘Quail callers’ do, however, also offer a tool for estimating quail abundance and developing more accurate population size estimates.

## Introduction

Hunting has been an integral aspect of human evolution and has co-evolved with human tool-use for hundreds of thousands of years [1–3]. Over this time, humans have continuously innovated their hunting techniques and technologies. Historically, this has involved the development of projectile weapons (e.g. arrow heads) [4] and the integration of domestic animals (e.g. hunting dogs) [5]. Modern developments have included the utilization of advanced technology such as non-lead firearm ammunition [6], thermal and night vision scopes [7], scent-neutralizing technology [8], trail cameras [9] and drones [10]. There is increasing scrutiny of newly developed technology applied to hunting and a growing societal expectation that empirical testing of outcomes related to sustainability or animal welfare will occur, in order for social license to be maintained [6].

Deception is a key strategy used by hunters to gain proximity to their target species, particularly using lures to draw in or aggregate animals [11, 12]. Lures include olfactory cues used in traps [13], visual cues such as decoys [14], and acoustic lures such as whistles and imitated calls to attract hunted species [15]. Some lures have proven ‘too successful’ at aggregating hunted species and have subsequently been banned for use in recreational hunting in some post-industrial nations. For example, ‘spinning-wing decoys’ used for duck hunting have been banned in some North American jurisdictions [14]. Recently, there has been a wide uptake by hunters of Electronic Acoustic Lures (EAL) which broadcast the vocalizations of a target species as an attractant [16, 17]. There are, however, considerable concerns about the direct (target species) and indirect (non-target species) impacts of the use of EALs in hunting on sustainability, with attendant ethical concerns.

EALs may increase a hunter’s harvest rate far beyond what has been historically possible, especially where the hunted species are cryptic, widespread at low densities, or both. For example, lesser snow geese (*Chen caerulescens caerulescens*) were found to be highly vulnerable to EALs in the USA [18]. The use of EALs has the potential to reduce the sustainability of hunting due to their “deadly effectiveness” [19], and their use is heavily regulated or prohibited in many countries [19]. Traditional hunting has spiritual ties where the harvest is not a measure of success whereas modern hunting is more experiential and opportunistic where success is determined by some on the size of the harvest [20]. The Eurocentric concept of ‘fair chase’ has been adopted in much of the western world as a moral standard by which hunters allow game a reasonable fighting chance [21] and is embedded in various legislation and regulation involving hunting [22]. If effective, EALs have the potential to significantly compromise the ‘fair chase’ ethic of hunting. EALs may also lead to indirect effects such as attracting and concentrating predators of the target species, further compromising sustainable hunting practices.

Predators also have the capacity to learn and therefore exploit the luring effect of ‘quail callers’ over time [23–25]. EALs may therefore facilitate the creation of an ecological trap, attracting prey to areas in high density where predators are also attracted and increasing the hunting efficiency of both hunters and predators. A fundamental assumption of the concerns associated with EALs is that they are in fact effective in attracting and concentrating their target species, yet there is a critical lack of literature evaluating EALs in a hunting context.

Stubble quail (*Coturnix pectoralis*) are a small (~100 g), cryptic, ground-dwelling grassland galliform species [26]. It is considered to be the most common quail species in Australia and is found in all states and territories, except Tasmania. Stubble quail are generally found in habitats of minimal to no canopy cover, such as agricultural lands and grasslands [27, 28]. On mainland Australia, the species’ range and population is believed to have expanded following the clearing of forest and woodland and the establishment of crops and irrigated agricultural lands [27]. Prior to European settlement, their preferred habitat was native grasslands, however today, much of this habitat type has been removed or highly degraded in Victoria [29]. While the stubble quail is well adapted to agricultural areas, improvements in farming practices, such as the widespread use of pesticides and use of expansive crop monocultures, may be detrimental to their success by removing food, habitat complexity and cover for much of the year [30]. This has been found in other ground-dwelling gamebirds in other parts of the world [31, 32], but is not well understood in Australia. Stubble quail movements and breeding are highly influenced by rain and flood events and resultant food availability [33] and they exhibit nomadic behaviour, being capable of long-distance dispersal [33]. They display boom-and-bust abundance cycles [34] with a regular spring/early-summer breeding period and frequent second breeding known to occur in late-summer/autumn if conditions are favourable [28, 35]. Little is known of their social organisation or behaviour in the wild, including their breeding behaviour, and whether they establish territories, including during the breeding period [29].

The stubble quail is the only native ground bird that can be hunted in the south-eastern Australian states of Victoria and South Australia [26]. In Victoria, they are hunted using shotguns and gundogs [26], mostly on privately owned agricultural land where cropping or grazing is the primary land use and have been hunted in this way for over 100 years [34, 36]. In Victoria, the stubble quail hunting season extends from the first Saturday in April to the end of June, inclusive, each year (the hunting season) [34], with a strict daily bag limit (20 birds per hunter per day). There are no sex-based restrictions within this. Total seasonal harvests can vary from the tens of thousands to the hundreds of thousands as populations fluctuate in response to seasonal conditions [34, 37].

The use of EALs (commonly referred to as ‘quail callers’) is currently legal for quail hunting in Victoria, with the use of the technology increasing rapidly [38]. While uptake of ‘quail callers’ has increased, there is no literature available that currently assesses the effectiveness of EALs in attracting stubble quail. We, therefore, aim to investigate the efficacy of the EAL ‘quail callers’ in attracting and concentrating stubble quail. Further to this, we also examine whether ‘quail callers’ have the potential to lead to indirect effects by attracting predators of stubble quail.

## Methods

### Ethics statement

This research was supported by the Deakin University Animal Ethics Committee (AEC) approval #B33-2020 and in accordance with Department of Environment, Land, Water and Planning Wildlife Act Research Permit No. 10009735.

### Study area

**Fig 1:**
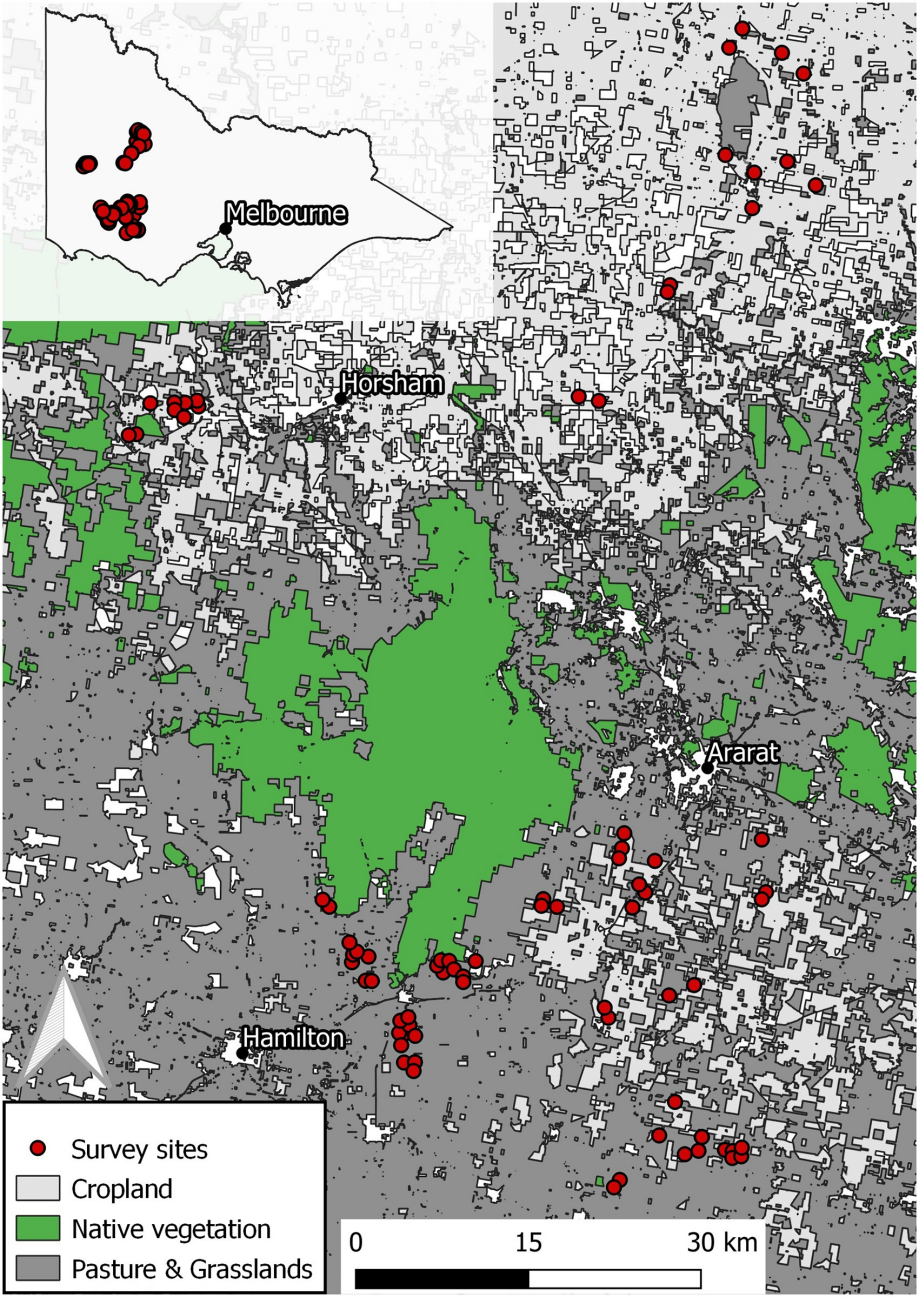
Map of sites used to assess the effectiveness of ‘quail caller’ Electronic Acoustic Lures (EALs) to attract stubble quail (*Coturnix pectoralis*) on private properties in western Victoria, south-eastern Australia, 2021.

This research was undertaken in western Victoria, Australia, a primary production district with vast areas of privately owned agricultural land representing key habitat for stubble quail [28, 33, 39]. Stubble quail are commonly hunted throughout the study area during the hunting season. Surveys were conducted at various locations in the Buloke, Yarriambiack, Horsham, Southern Grampians, Ararat, Moyne, and Northern Grampians regions (see Fig 1). The daily average rainfall across western Victoria increased during the study period from 1.43 mm per day in May and 3.34 mm per day in July), and the daily average temperatures dropped (maximum daily average of 13.9°C in May, to 12.4°C in July).

### Study design

Surveys were conducted during the hunting season of 2021 to establish the effect of ‘quail callers’ during the period when they are actually used in hunting. Seventy-nine sites on private land were selected across a range of habitat types including pasture crop (N = 27 sites), cereal and legume stubble (N = 21), native grasslands (N = 19), low open woodlands (N = 5), regenerative cover crops (N = 3), freshwater marshland (N = 2), and rocky outcrops (N = 2). Sites chosen for the study have been subjected to differing levels of agricultural disturbance (magnitude, depth and intensity of human intervention), from minimal or no disturbance (e.g., low open woodland) to consistent heavy cultivation (e.g., chemically fallowed ploughed stubble). Sites were selected based on three key criteria: 1) they had confirmed recent or historical presence of stubble quail; 2) our sampling would not impede agricultural practices; and 3) agricultural practices would not influence the presence of stubble quail. Sites were maintained at least 1 km apart to increase independence (mean±SE, 2.45 ± 0.02 km).

**Fig 2:**
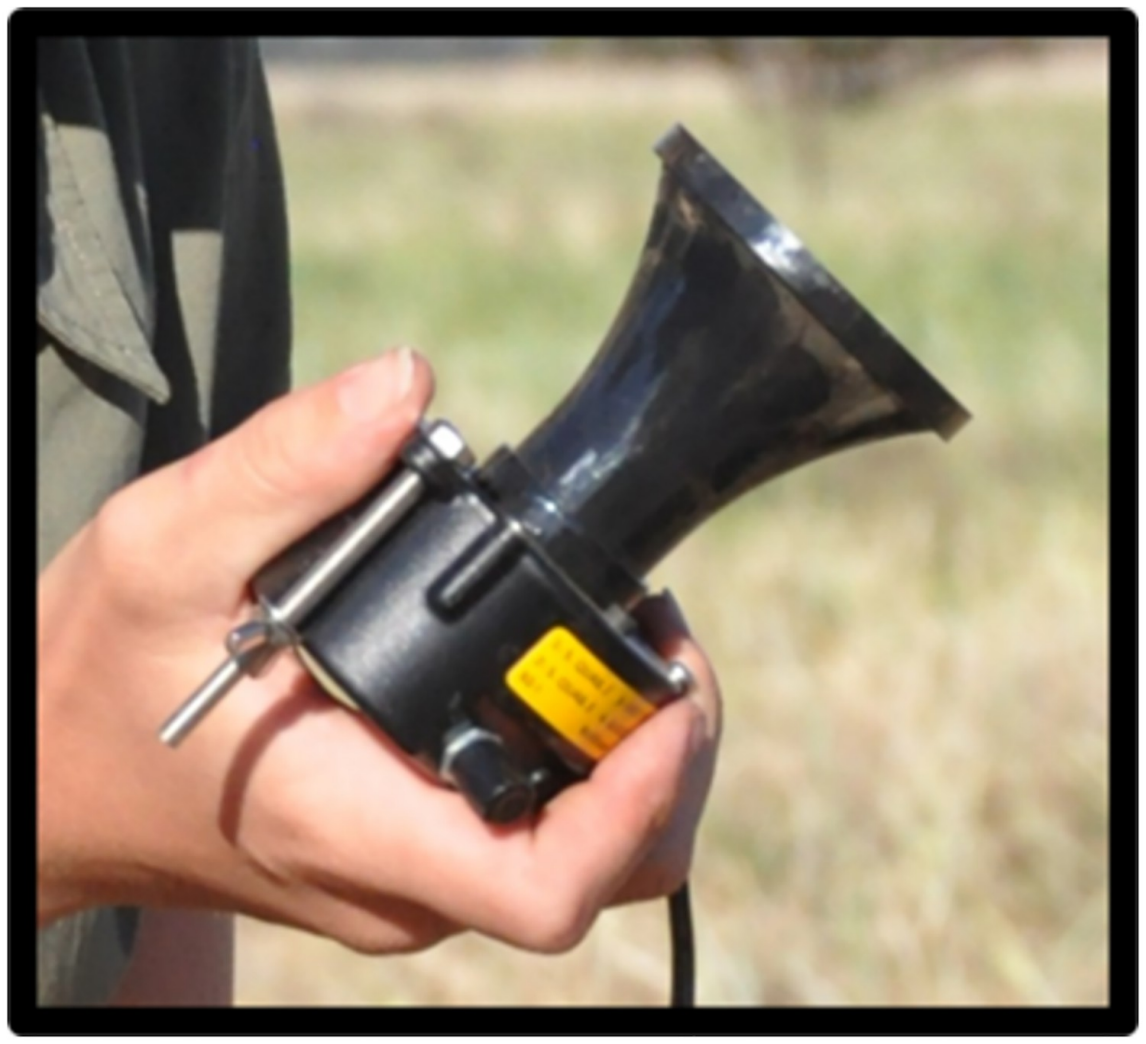
Photo of a QG-25 ‘quail caller’ Electronic Acoustic Lure (EAL) used to attract stubble quail (*Coturnix pectoralis*) on a private cropping property in south-eastern Australia, 2021.

At each site, a matched paired design was used where two 200 m transects, positioned in a north-south orientation, were established 300 m apart. Each transect was randomly assigned as either ‘active’ (i.e. a site where a ‘quail caller’ was deployed and activated), or ‘control’ (i.e. a site where a ‘quail caller’ was deployed but inactive). The paired nature of this design enabled us to account for differences in habitat type, climatic and weather conditions throughout our surveys. A ‘quail caller’ (QG-25 Quail Caller, Multisound, Italy) [40] was deployed at the center point of each transect. ‘Quail callers’ were 260 g in weight, 10 cm long × 6 cm in diameter and were connected to a 12 V 6.6Ah battery (Fig 2). ‘Quail callers’ were mounted on wooden stakes at a height of ~ 300 mm above ground. The ‘quail caller’ used a recording of a male stubble quail, thought to be an advertisement of nesting territory made during the reproductive period, July–January [40]. While a quail caller could be heard over distances of 300–400 meters (depending on weather conditions) the matched pairs design used here allowed us to establish the effect of our treatments within a site. The distance between site pairs of over 1km (mean 2.45km) was however beyond the range at which callers could be heard.

Distance sampling was conducted during daylight hours only and at 72% of sites (N = 57, sampling time: 5.46 ± 0.19 (SE) minutes at ~2.20 km/h) as it is known to be effective at estimating abundance for other species of quail [17, 41–43]. Each transect was surveyed once prior to turning the ‘quail callers’ on to establish a baseline estimate. The ‘quail caller’ at the ‘active’ treatment was then turned on and left active for 48 hours continuously night and day after which both transects were resampled. This time interval was chosen to minimize habituation to the call [44] and because it mimics the approximate time hunters leave quail callers active prior to hunting. For each stubble quail detected during surveys, the number of individuals, location along the transect, angle of observation relative to the transect line, and distance to the quail from the transect were recorded, as per standard convention for walking distance sampling [42].

At the center point of each transect an infrared trail camera (ScoutGuard SG550V, ScoutGuard, Gold Coast, Australia) was also deployed facing the ‘quail caller’, 20 m away. Cameras were set approximately 30 cm above the ground and were set on high sensitivity to take three consecutive images when triggered. Cameras were left *in situ* for two days and placed in a SE–SW direction. Cameras were initially used to establish a metric of stubble quail activity, although were quickly replaced with distance sampling when stubble quail detections were absent even though they were known to be in the area. Cameras, however, also enabled the assessment of non-target predators that may have been attracted to ‘quail callers’ [24, 45]. Cameras were programmed to take three consecutive photographs with a 15 s interval between triggers.

### Data analysis

We first used a Generalised Linear Mixed Model (GLMM) in ‘lme4’ [46] in the statistical program ‘R’ [47] to determine the detectability of stubble quail between time periods (before and after the ‘quail caller’ was activated), treatment (active and control transects) and an interaction between the two. We used presence/absence data along each transect resulting in a binomial distribution. We included a random effect of site to account for repeated measures; however, we were unable to include a nested random effect of time period within site due to a lack of replication. This omission increases the likelihood of a type II error (i.e. falsely accepting the null hypothesis). We validated our model using visual assessments of residual values compared against fitted values, and residual values compared to each variable within the model.

To investigate whether the relative abundance of quail differed between the treatments we used distance sampling in the ‘Distance’ package in ‘R’ [48]. We first compared five detection functions (half normal, hazard rate, uniform with a cos adjustment, uniform with a polynomial adjustment, uniform with a hermite polynomial adjustment) and used Akaike’s Information Criterion (AIC), in combination with a visual assessment, to determine the most appropriate detection function. Due to an absence of within-survey replication, we were unable to consider transect-level estimates of relative abundance, instead we estimated these in two ways: 1) compare the two pre-activation transect surveys to the two post-activation transect surveys (i.e. pooling across treatments), and 2) comparing the two repeated transects at the control treatment to the two active transects (i.e. pooling across surveys). To generate estimates of abundance we specified the sampling region as 1.2 ha (200 m transect × 60 m detection range), given the nature of our surveys these metrics should be interpreted as a relative abundance that is comparable across treatments, but may not reflect true abundance at those sites sampled.

Detections on cameras were sorted to identify potential predators of stubble quail which may have equally been attracted to the ‘quail callers’. We defined a predator event as an image of a predator (e.g. red fox *Vulpes vulpes*) that was at least 15 minutes apart from another image of the same species. We compared the detections of predators (presence/absence) to each treatment (control/active) using a Chi-squared test of independence using a continuity correction.

## Results

**Fig 3:**
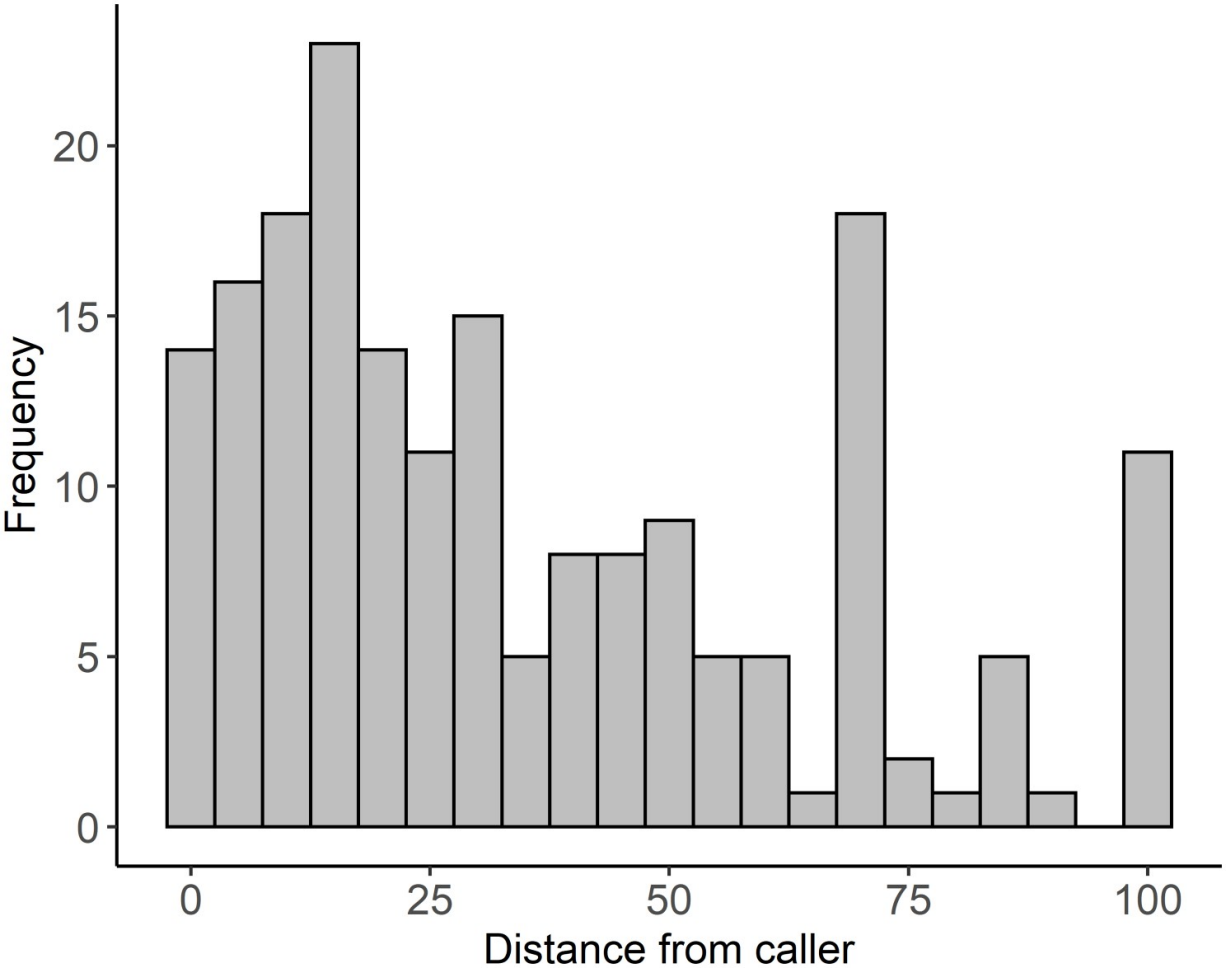
Frequency of quail detection with respect to the distance from the ‘quail caller’ (10 m increments).

A total of 228 transects were surveyed detecting 508 stubble quail. Thirteen quail were detected during pre-treatment surveys (62% at control, 38% at active transects); 495 stubble quail were detected during the post-treatment surveys 99% of which were at active sites. When stubble quail were detected at active sites, we found they were more likely to be detected closer to the location of the ‘quail caller’ (Fig 3). It was not possible to record the age or sex of every detected stubble quail given the distance and speed at which they flushed. Red foxes were the only predator detected more than once on cameras as potential predators of quail, detected at 11% of sites with no difference in detection between treatments observed (χ^2^ = 0.615, df = 1, p = 0.433).

**Fig 4:**
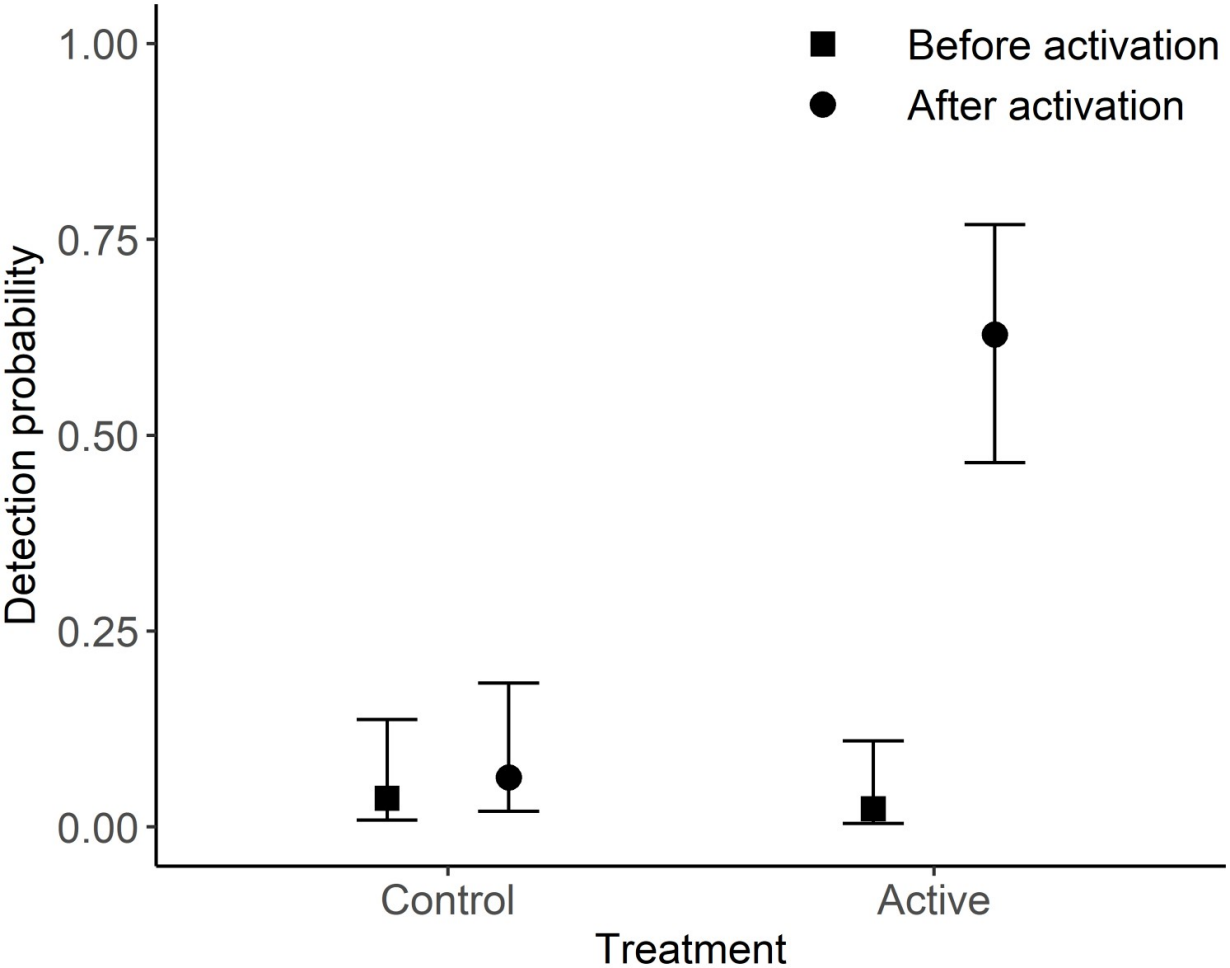
The detectability (mean ± 1.96SE) of stubble quail during pre- and post-transect surveys at both the control (‘quail caller’ inactive) and treatment (‘quail caller’ active) sites.

The presence of quail at a transect was influenced by treatment (β = 4.152, 95%CI: 0.165–8.140), the time period (β = 4.265, 95%CI: 2.438–6.091) and the interaction term between the two (β = -3.687, 95%CI: -6.037–1.336). The probability of detecting quail at a site during pre-surveys was 0.023 (95%CI: 0.005–0.110) and 0.037 (95%CI: 0.010–0.137) at active and control sites respectively. This increased during post-treatment surveys with the probability of detecting quail at active sites (β = 0.629, 95%CI: 0.465–0.769) being considerably higher than at control sites (β = 0.063, 95%CI: 0.020–0.184; Fig 4). This model had good explanatory power with 40% (R^2^_m_ = 0.401) of the data explained by the model fixed effects and 54% explained by the full model (R^2^_c_ = 0.536).

**Fig 5:**
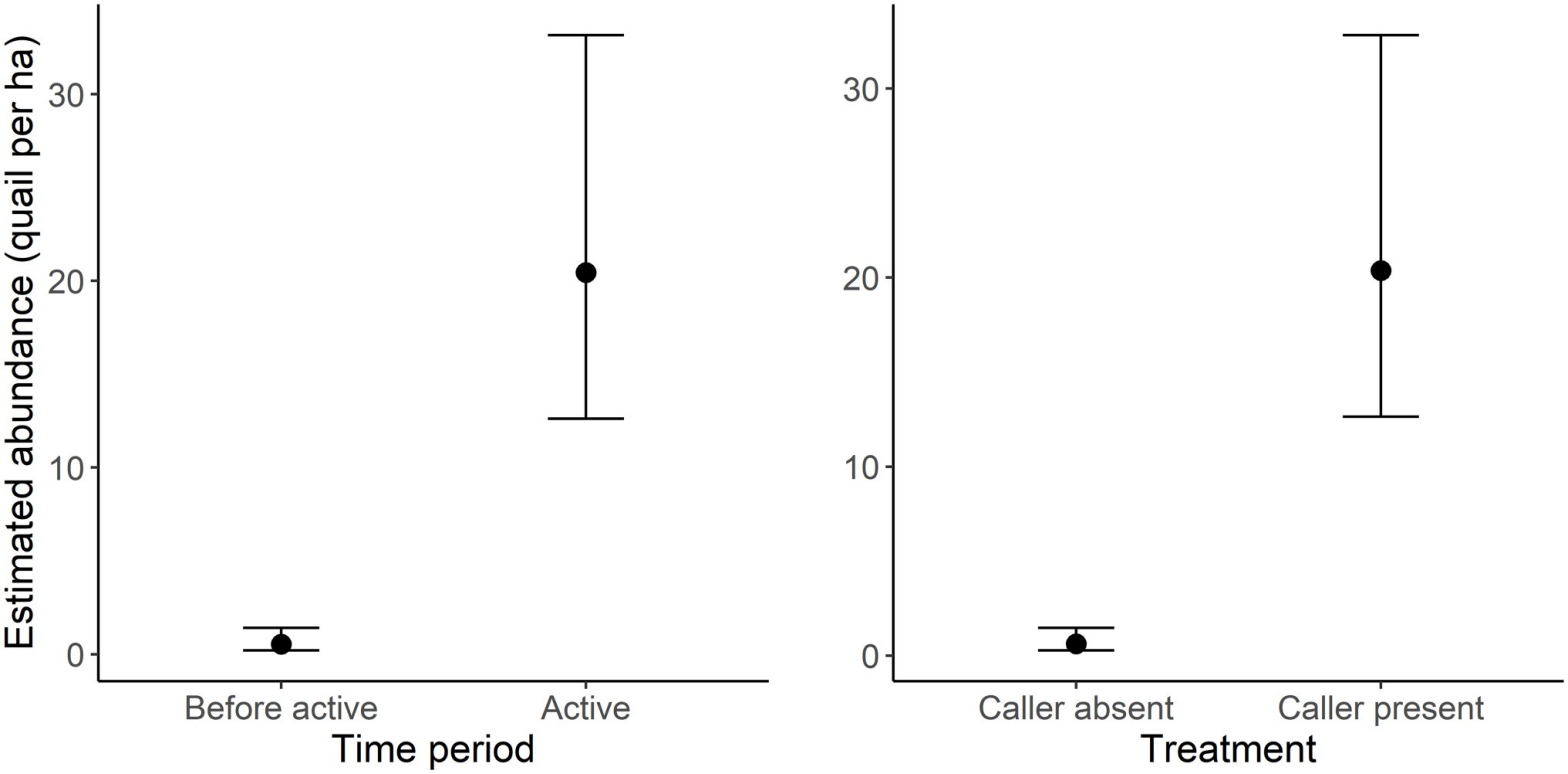
Estimated quail abundance (mean ± 1.96SE) between time periods (with treatments pooled) and treatments (with time periods pooled).

The best supported detection function for distance sampling was a hazard rate which was well supported over a half normal (ΔAIC = 5.16), and all uniform detection functions (cos: ΔAIC = 8.45, polynomial: ΔAIC = 32.29, hermite polynomial: ΔAIC = 159.77). This detection function demonstrated that most quail were detected within 10 m of the transect, declining sharply towards 20 m from the transect (S1 Fig). Using this detection function, relative quail abundance was estimated to be lower during pre-surveys (0.537, 95%CI: 0.204–1.417) when compared to post-surveys (20.456, 95%CI: 12.616–33.168). Quail abundance was also estimated to be higher on active transects (20.373, 95%CI: 12.644–32.829) when compared to control transects (0.631, 95%CI: 0.272–1.461; Fig 5).

## Discussion

Electronic acoustic lures have proven to be an effective tool for attracting birds in a variety of environments [23, 49–51]. These findings are valuable when applied to research methods for studying wildlife but are of concern for sustainability when the technology is used to harvest animals. Our results indicated that the probability of detecting stubble quail at sites with active ‘quail callers’ was an order of magnitude higher than at control sites. The significant increase in stubble quail detections and abundance/density at transects with active ‘quail callers’ supports the hypothesis that the devices are effective in attracting stubble quail. This increase was in the context of prevailing low rates of detection and abundance (i.e. before trials) suggesting that the abundance of stubble quail occurring naturally across all study sites was low and dispersed across the landscape. Not only were stubble quail attracted to active ‘quail callers’, but they were most frequently detected within 30 m of the active caller. Thus, not only do ‘quail callers’ have a strong effect on the local abundance of stubble quail in response to active callers, but we demonstrate the ability of the devices to concentrate stubble quail into a very localized area, and over a relatively short time frame (48 hours).

Degree of attraction to EALs may vary with duration of exposure to calls [52, 53]. Thus, in line with studies of other EALs, the capacity to draw birds close to the device [44], for considerable amounts of time [52], seems likely, and potentially facilitates ease and efficiency of hunting. The prerequisite for potential problems with the use of ‘quail callers’ for stubble quail–they offer a practical and effective tool to increase hunting efficiency (as for lesser snow geese [18])–is clearly evident. This likely makes ‘quail callers’ attractive to hunters as a tool to increase harvest success, although the relationship between hunter success rates, and frequency of hunting trips (i.e. hunting effort) [34], and ‘quail caller’ use remains unknown. Based on the findings in this study, there is the real possibility that ‘quail callers’ could increase the ability for less skilled hunters or those who hunt without dogs to increase their effectiveness and subsequent harvest. Already skilled and effective hunters could further increase their harvest and some hunters could be tempted to exceed the strict daily bag limits (20 birds per hunter per day) when confronted with significant concentrations of birds.

While annual total harvest levels are known in Victoria [30, 31], their relationship to total population abundance–and temporal changes in abundance–is not and technologies such as ‘quail callers’ that have the potential to increase harvest levels may threaten the sustainability of hunting. This highlights the need for a greater understanding of the stubble quail population in the face of advancements in technology which may increase hunter success. At the time of our study, no landscape-scale abundance monitoring was being implemented. However, a program to survey stubble quail abundance in Victoria commenced in January 2022 as part of a state government initiative to ensure sustainable hunting [54]. Robust abundance estimates together with the existing program of monitoring for quantifying trends in harvest levels and catch per unit effort [34] will provide important data to ensure that hunting is sustainable.

Red foxes were the only possible predator of stubble quail to be detected more than once. Data were too few for a robust test of whether foxes are attracted to active ‘quail callers’. However, this does not mean that there is no response from predators to the devices–no detection does not mean that there was nothing to be detected [55]. Additional sampling is required to robustly test whether foxes are attracted to ‘quail callers’, and the enhanced probability/density of stubble quail which occur near them. Additionally, the possibility of repeated use of ‘quail callers’ within a fox’s home range also means that attraction may occur over time, perhaps associated with learning on the part of the predator [23–25, 56]. We also note that the treatments were only 300 m from each other and, as such, foxes could also have responded to the visual stimuli of the control treatment near the active treatment.

The use of EALs, given their effectiveness, may provide an effective survey tool for assessing quail abundance and distribution, and underpin enhanced understanding of habitat usage, and landscape-scale features that correlate with stubble quail occurrence. Paradoxically, something that has the potential to compromise the sustainability of hunting may also provide a tool to develop more informed estimates of population size and therefore sustainable hunting regulations based on known population sizes.

In addition to their regular spring-summer breed, stubble quail are known to breed in late-summer and autumn if conditions are favorable [28, 35]. This coincides with the hunting season and when this study was conducted. This could explain, at least in part, the success of the ‘quail caller’ used in this study which played a male call considered to advertise nesting territory [29]. It is feasible that there was a phenological effect linked to the male territorial call being used during a time of the year when breeding can occur. This would likely attract females looking for a mate and / or competing males. Whether female calls played from ‘quail callers’ would have the same success in attracting large numbers of stubble quail is unknown and would require further investigation.

It was not possible in this study to determine whether stubble quail were attracted to the caller solely in response to its aural stimulus or whether visual or other stimuli also had an effect. It is possible that the sight or behavior of nearby conspecifics may have caused a ‘flocking effect’ [57] which influenced other stubble quail to follow attracted birds towards and concentrate in the vicinity of the caller. We were not able to consistently identify the age or sex of flushed birds to determine whether particular cohorts were attracted or whether the attraction was due to direct (aural) or indirect (e.g. flocking) causes, or both. This would require further investigation with careful experimental design. Potential sex bias in attraction to EALs might amplify the population impacts of hunting stubble quail over time [28], indeed sex-specific EALs are available for some bird species [58]. The ‘quail caller’ model used in this study deployed a male call and it would be of interest to see if this was attractive to females or competing males. If ‘quail callers’ disproportionately attract females, then the sustainability of the devices in hunting could be further compromised.

We demonstrate that ‘quail callers’ concentrate and aggregate stubble quail and as such have the potential to markedly increase the harvest rate of stubble quail, compromise the concept of fair-chase, and may cause indirect impacts, such as disturbance from foraging and other behaviors, that direct energy into responding to the ‘quail callers’–an area requiring further research. Consideration should be given to regulating their use, particularly in the context of a lack of rigorous stubble quail abundance estimates at present creating the potential for undetected unsustainable harvesting. ‘Quail callers’ could, however, if investigated further, be an advantageous tool for facilitating much-needed ecological research on this species.

## Supporting information

S1 File (CSV)

S1 Fig Detection probability of quail with distance (m) from the transect. (TIF)
