# Supplementary figures and images for: Assessing the efficacy of electronic quail callers in attracting stubble quail and non-target predators
Source: PLoS One. 2022 Jul 22;17(7):e0271893. doi: 10.1371/journal.pone.0271893 (PMC9307177; doi:10.1371/journal.pone.0271893)

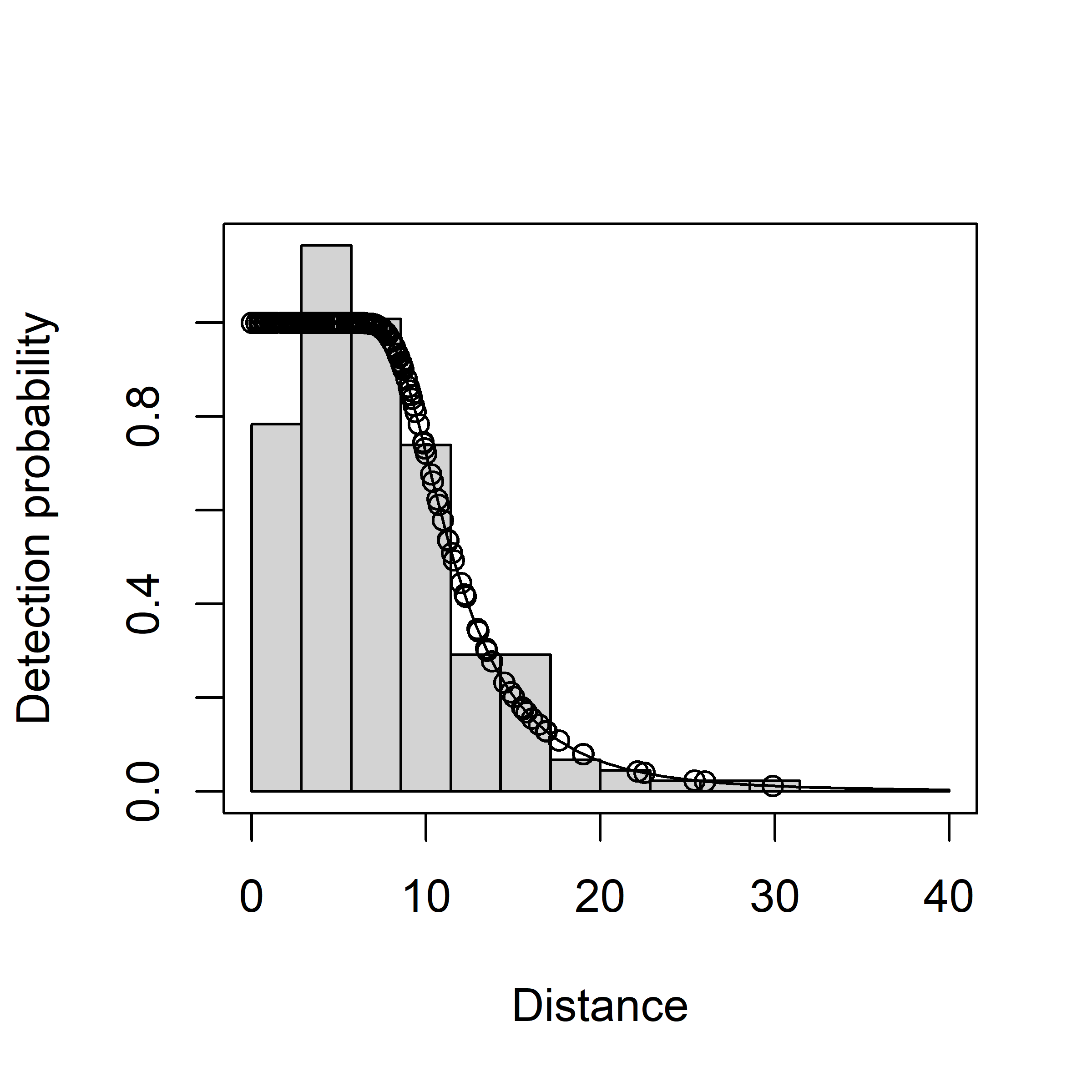

Supplement: S1 Fig — (TIF) [file pone.0271893.s002.tif]
